# Supplementary material for: Logical design of synthetic cis-regulatory DNA for genetic tracing of cell identities and state changes
Source: Nat Commun. 2024 Feb 5;15:897. doi: 10.1038/s41467-024-45069-6 (PMC10844330; doi:10.1038/s41467-024-45069-6)
Supplement: Supplementary file 1 — Supplementary Information [file 41467_2024_45069_MOESM1_ESM.pdf]

Supplementary Figure Legends

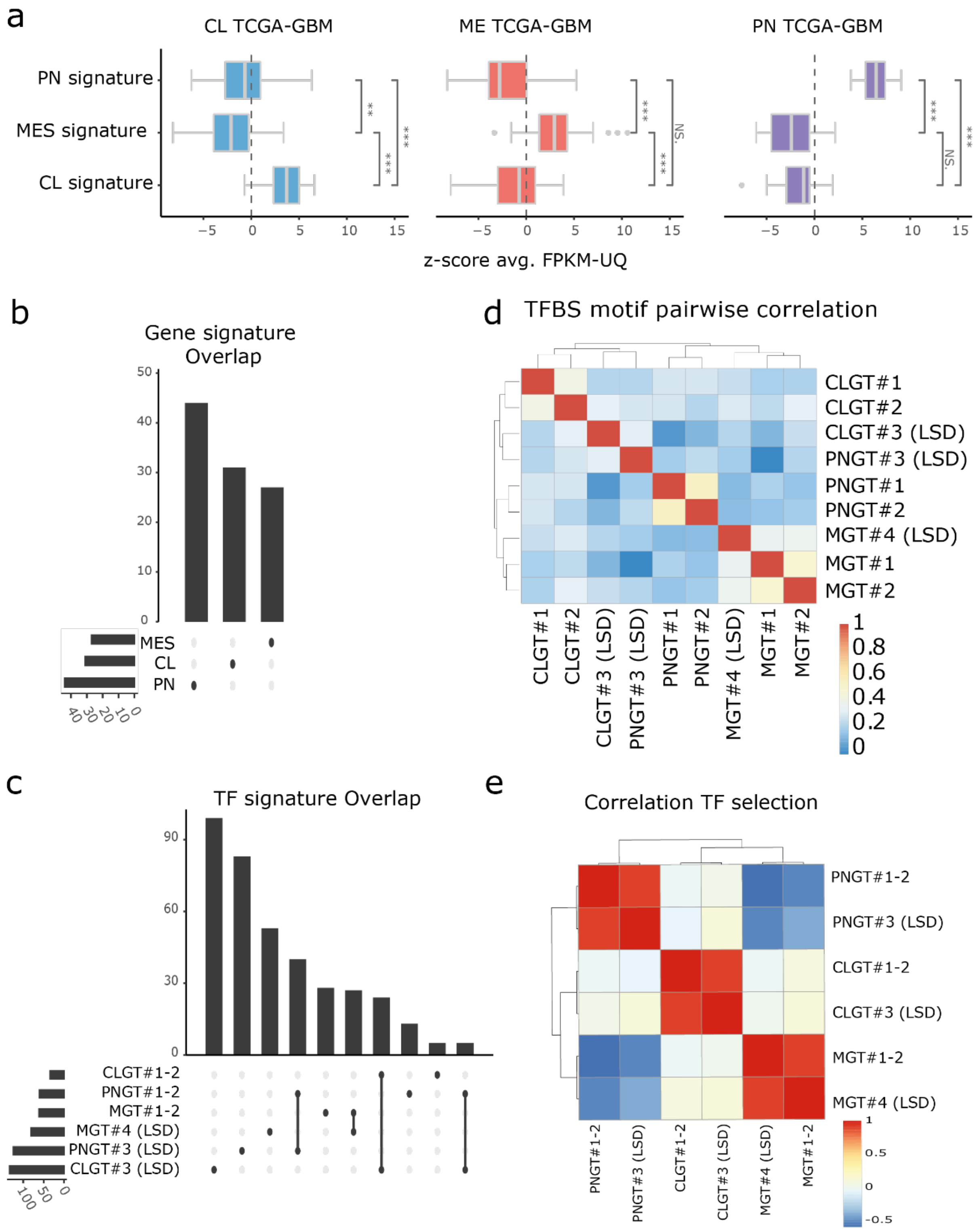

Figure S1 | Correlation analyses of LSD input signature and TF lists.

a) Boxplot comparing gene expression z-scores for the indicated sLCR's signature gene sets in glioblastoma patients (TCGA-GBM, FPKM-UQ). Each annotated GBM transcriptional subtype features statistical comparisons by two-sided pairwise t-test. Data distribution is shown, with box indicating the interquartile range and inner line indicating the median. Whiskers extend to represent the data range, including outliers. b) Upset plot depicting the number of and overlap between signatures genes underlying each 1st generation/LSD GBM subtype sLCR. c) Upset plot depicting the number of and the

overlap between TF underlying each GBM subtype sLCR designed by LSD. The connected lines denote the overlaps. d) Heatmap showing the Pearson correlation between the indicated input TFBS lists with respect to overall TFBS. Hierarchical clustering analyses used Euclidean distance and complete linkage. e) Heatmap showing the Pearson correlation of ssGSEA enrichment scores in Glioblastoma patients' expression for each TF input lists. Both hierarchical clustering analyses used Euclidean distance and complete linkage. sLCR = synthetic locus control region; GBM = Glioblastoma; LSD = logical synthetic cis-regulatory DNA; TF = Transcription Factor; TFBS = Transcription Factor binding site; MES = Mesenchymal; PN = Proneural; CL = Classical; ssGSEA = single sample gene set enrichment analysis. Source data are provided in the Source Data file.

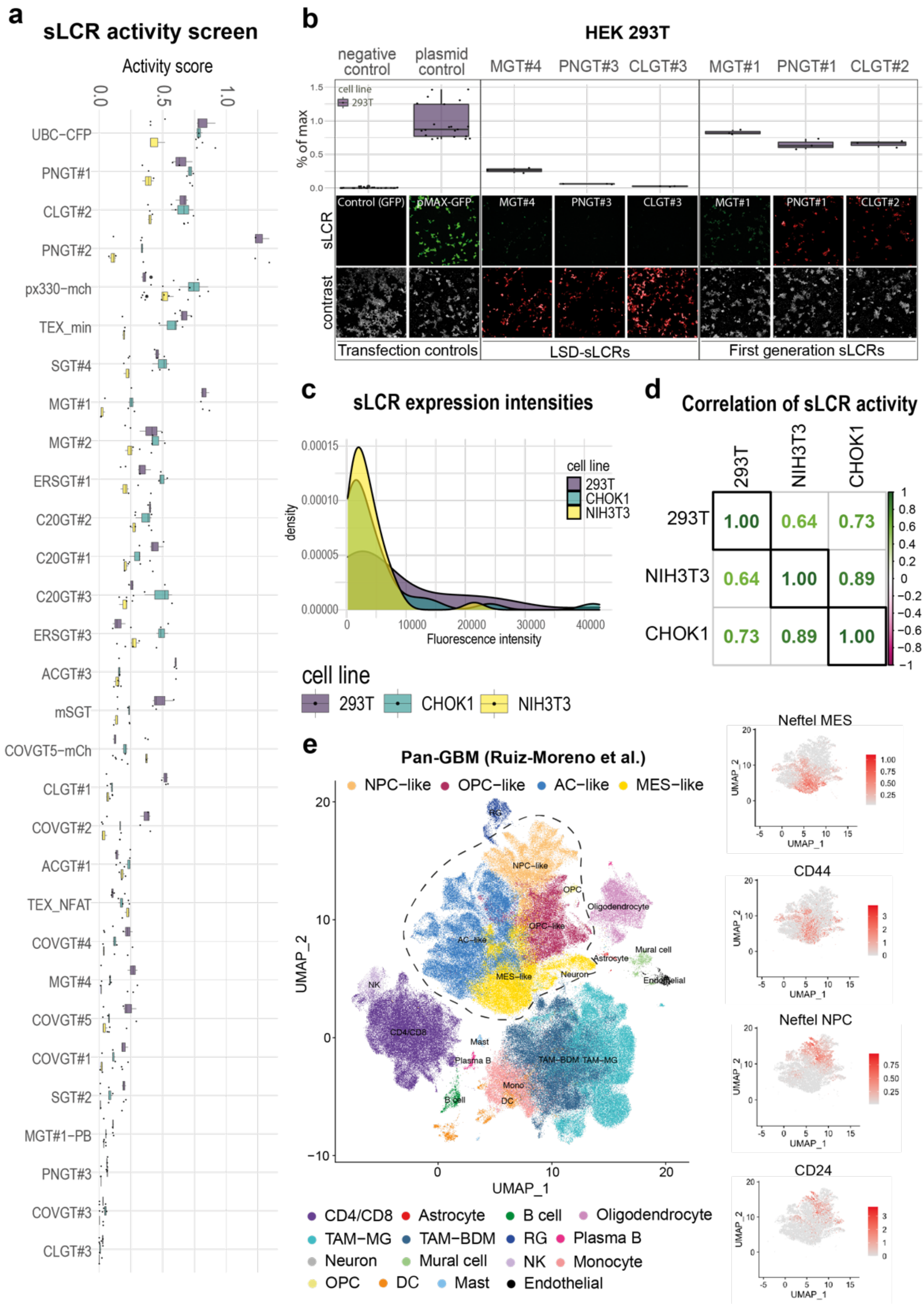

Figure S2 | Extended analysis of sLCRs desgned by LSD.

a) Box plot of indicated sLCRs (n=28) transfected in human epithelioid 293T (purple), hamster epithelioid CHO-K1 (teal) and mouse fibroblastoid NIH3T3 (yellow) cell lines. The top axis shows fluorescence normalized by controls and transfection efficiency per cell line. Each sLCR measurement was assessed in technical replica (n=3). Data distribution is shown, with box indicating the interquartile range and inner line indicating the median. Whiskers extend to represent the data range, including outliers. b) Upper, box plot comparison of activity scores for LSD and first generation sLCRs. Below, fluorescence microscopy images of sLCR expression (top panel) and contrast channel of either brightfield or independent promoter-driven fluorophore (lower panel). c) Density plot of raw fluorescence intensities of all datapoints for all sLCRs (n=28) in 293T (purple), CHO-K1 (teal) and NIH3T3

(yellow) cells. d) Pairwise pearson-correlation matrix of human and non-human cell lines for calculated sLCR (n=28) activities. e) Left, Uniform Manifold Approximation and Projection (UMAP) representation of the pan-Glioblastoma dataset from Ruiz-Moreno et al. [24] and corresponding color-coded cluster labels. Right, feature plot with enrichment scores for the Neftel GBM-signatures and CD44 or CD24 in the malignant cell compartment. Source data are provided in the Source Data file.

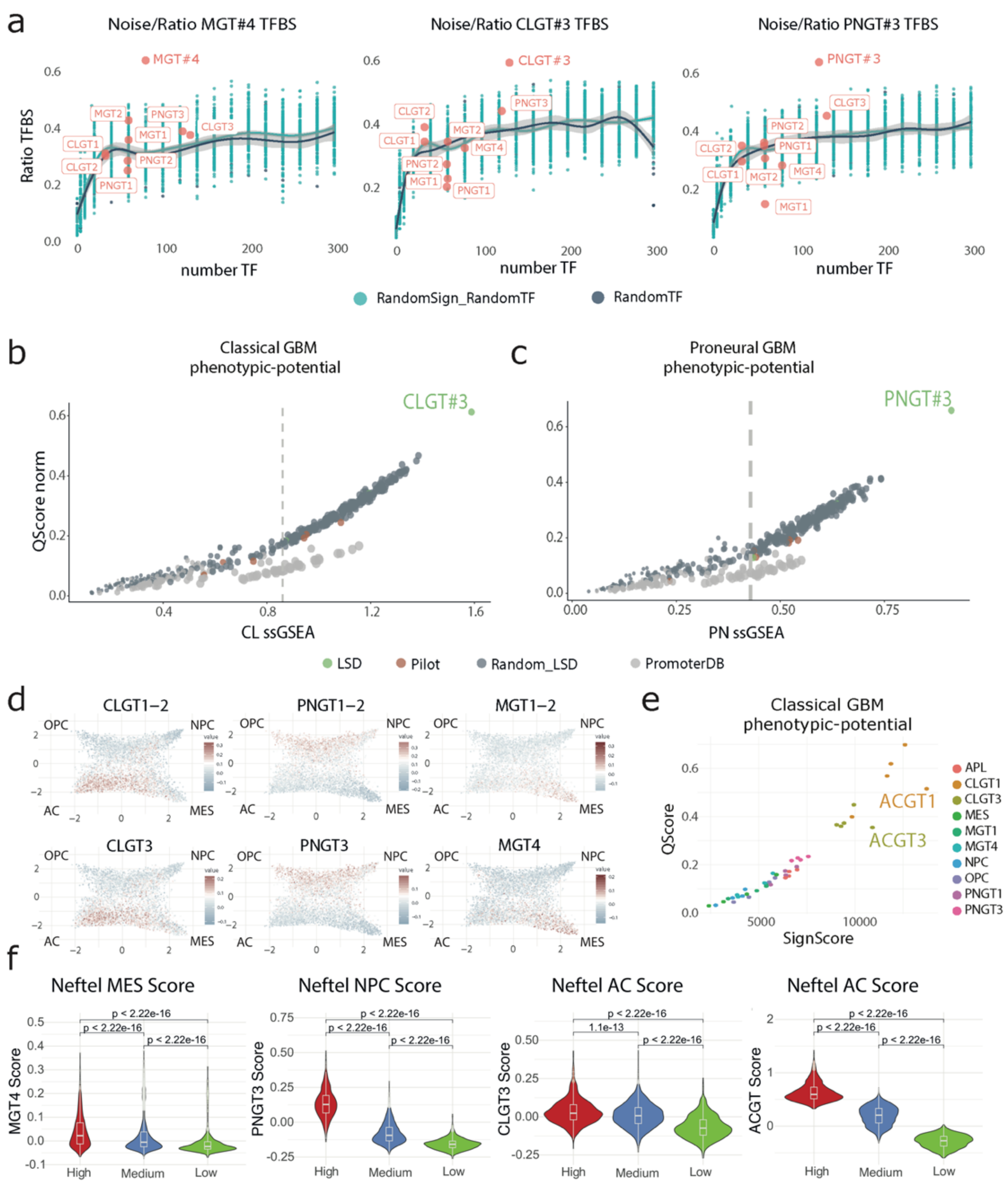

Figure S3 | Extended analysis of LSD sLCRs phenotypic potential.
a) Scatter plot showing the TFBS affinity ratio for on-target, off-target and randomly selected sLCRs. The Y-axis indicates the observed/expected ratio (i.e. observed/input TFBS) for the TFBS lists indicated in the header. The X-axis denotes the number of non-redundant input TF (see Methods). First-generation and LSD-designed sLCR are indicated in orange. Fitted lines indicate LOESS regression of sLCRs designed using LSD input from random sampling of TFBS on mesenchymal GBM signature genes (random TF; grey) or random signature genes (random Sign-TF; blue). b-c) Scatter plot showing the signature score (x-axis) and affinity score (y-axis; see Methods) of the indicated reporters (color-coded) for the classical and proneural phenotypes. d) ssGSEA normalized scores for signature genes for the indicated first generation sLCRs (above) and LSD-derived sLCRs (below). The cell states identified by Neftel et al. 2019[19] are indicated in each quadrant, and the original single-cell position is maintained

in the two-dimensional representation. e) Scatter plot showing the signature score (x-axis) and affinity score (y-axis; see Methods) of the indicated reporters (color-coded) for the classical phenotype. f) Violin plots of CD24/CD44 expression or LSD-sLCR signature gene scores for the indicated cohorts of patient-derived single cells[24] stratified according to the 15%-quantile highest (red), 70%-quantille medium (blue) or 15%-quantile lowest (green) module scores for signatures from Neftel et al.[19]. Data distribution is shown, with box indicating the interquartile range and inner line indicating the median. Whiskers extend to represent the data range, including outliers. P-values were calculated by two-sided t-test. TF = Transcription Factor; TFBS = Transcription Factor binding site; MES = Mesenchymal; NPC = Neural Progenitor Cell; AC = Astrocyte; OPC = Oligodendrocyte Progenitor Cell; ssGSEA = single sample gene set enrichment analysis. Source data are provided in the Source Data file.

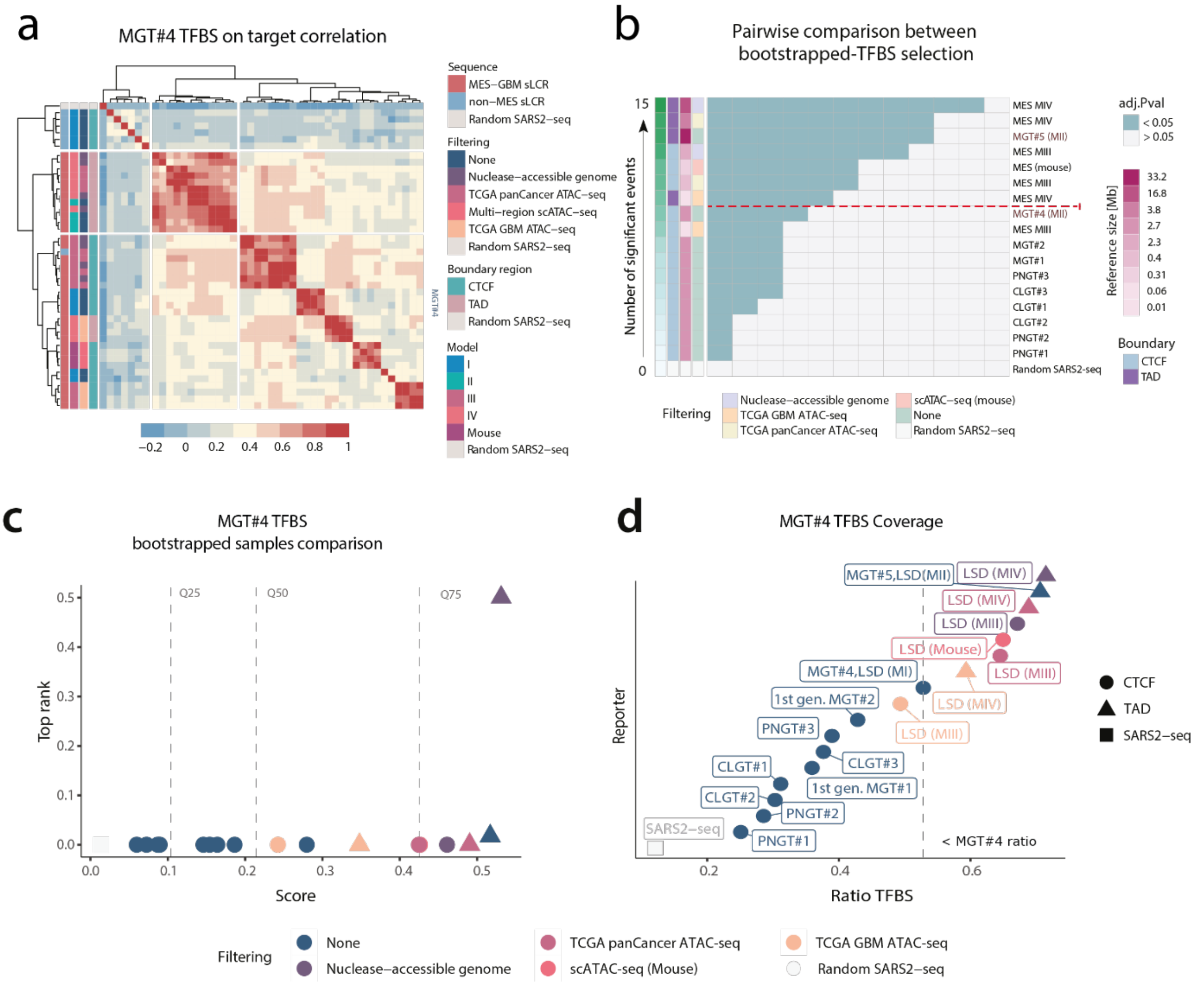

Figure S4 | Extended analysis of mesenchymal GBM sLCRs generation with custom inputs.

a) Heatmap showing Pearson correlation between the MGT4 TFBS score of the indicated sLCRs. Hierarchical clustering used Manhattan distance. b) Heatmap showing the significance of pairwise comparisons between the indicated sLCRs after bootstrapping of the MGT4 TFBS input (see methods). Color-code annotations denote the number of significant pairwise correlations between distributions (P.adj < 0.05), the boundary annotation method (i.e. nearest-neighbour CTCF or annotated TADs), the type and size of reference genome/subset used for TFBS mapping. The color-coding for the size of the genome input is also indicated. Dotted line marks threshold for models with improved number of significant events. c) Scatter plot visualisation of the MGT4 TFBS bootstrapped samples ranking based on the number of significant pairwise correlations between distributions (see Methods). d) Scatter plot visualisation of MGT4 TFBS coverage. Axis represents the observed/expected TFBS ratio (x-axis) of the indicated reporters (y-axis). Dashed lines highlight the threshold of MGT4 TFBS ratio. TFBS = Transcription Factor binding site; CTCF = CCCTC-binding factor; TAD = Topologically Associating Domain; ATAC-seq = Assay for Transposase-Accessible Chromatin sequencing. Source data are provided in the Source Data file.

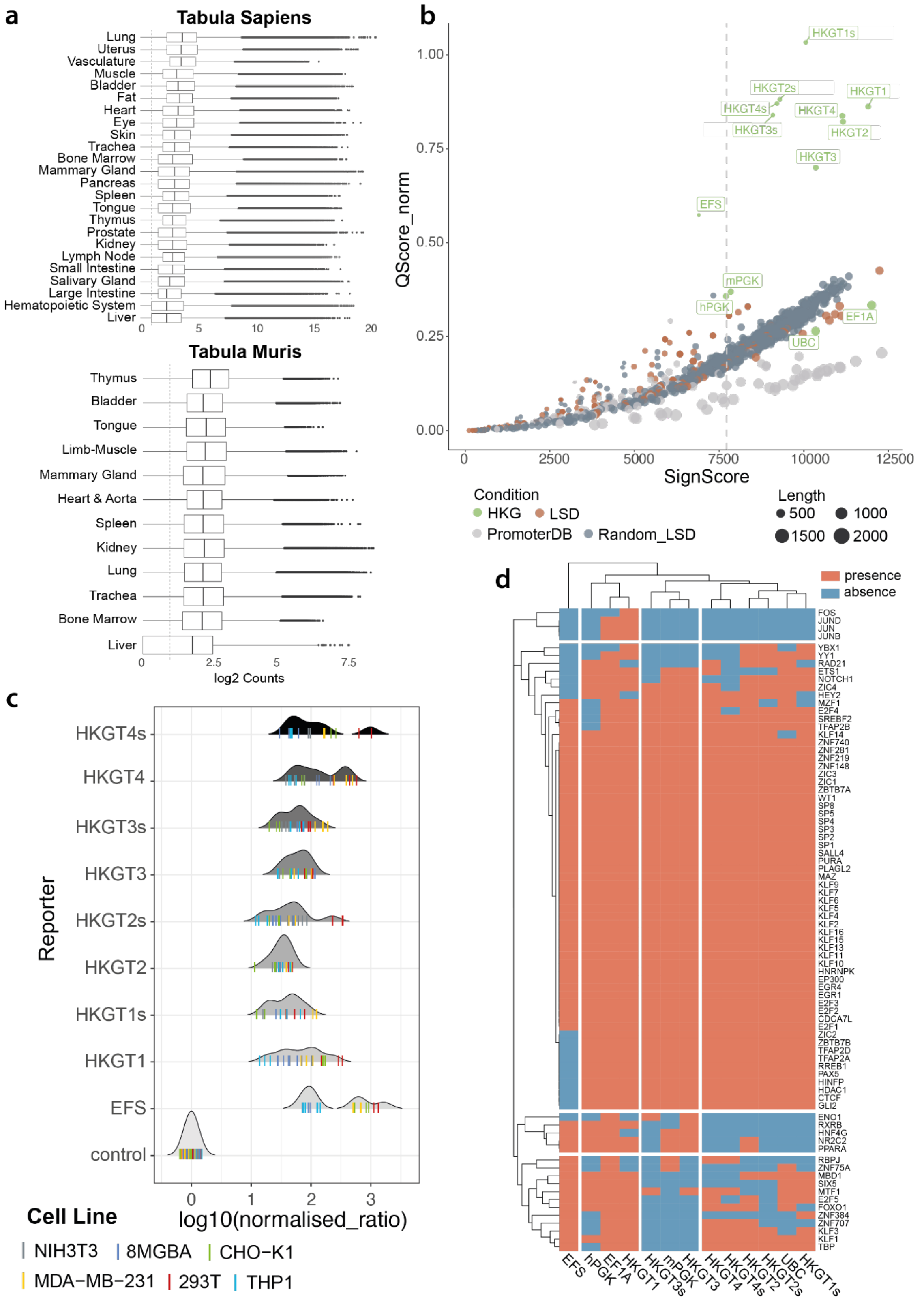

Figure S5 | Extended analysis of housekeeping-like sLCRs.

a) Boxplot comparing gene expression z-scores for specified housekeeping gene (HKG) sets in the Tabula Sapiens and Tabula Muris single-cell profiles. The line signifies the threshold below which 25% of total expression is observed in each data sets. Data distribution is shown, with box indicating the interquartile range and inner line indicating the median. Whiskers extend to represent the data range, including outliers. b) Scatter plot showing the signature score (x-axis) and affinity score (y-axis; see Methods) of the indicated reporters (color-coded). The dashed line represents the median of all

SignScores for that phenotype. c) Ridge plot showing log10-normalised mCherry intensity distribution for HK sLCRs and EFS promoter across all tested human, mouse (NIH3T3) and hamster (CHO-K1) cell lines (color-coded). d) Heatmap for absence or presence of indicated TFBS within the HKsLCR or selected promoter sequences. HKsLCRs = housekeeping-like sLCRs. Source data are provided in the Source Data file.

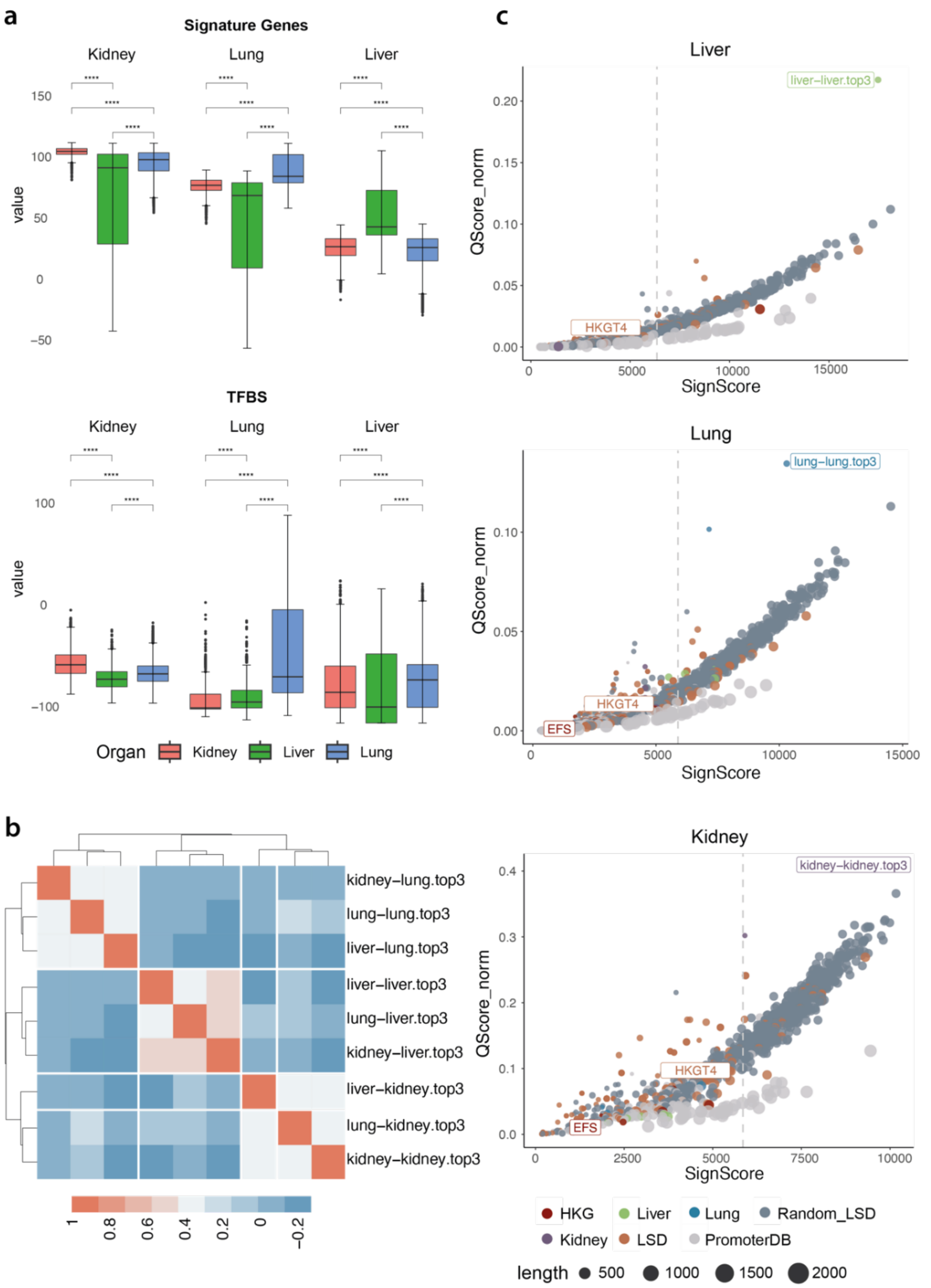

Figure S6 | Tissue specific design and in silico evaluation of tissue-specific sLCRs for gene therapy compatible with AAV-vectors size constraints.

a) Box plot presenting single-sample Gene Set Enrichment Analysis (ssGSEA) enrichment scores within the Tabula Sapiens dataset for the specified reporter signature and TF genes. Each color-coded specified tissue dataset transcriptional subtype is compared using the Wilcoxon rank-sum test, with resulting adjusted p-value < 0.05. Data distribution is shown, with box indicating the interquartile range and inner line indicating the median. Whiskers extend to represent the data range, including outliers. b) Heatmap showing the Pearson correlation between the TFBS score/diversity for each sLCRs-input TF list. c) Scatter plot showing the signature score (x-axis) and affinity score (y-axis; see Methods) of the indicated reporters (color-coded). The dashed line represents the median of all SignScores for that phenotype. TF = Transcription Factor; TFBS = Transcription Factor binding site; ssGSEA = single sample gene set enrichment analysis. Source data are provided in the Source Data file.

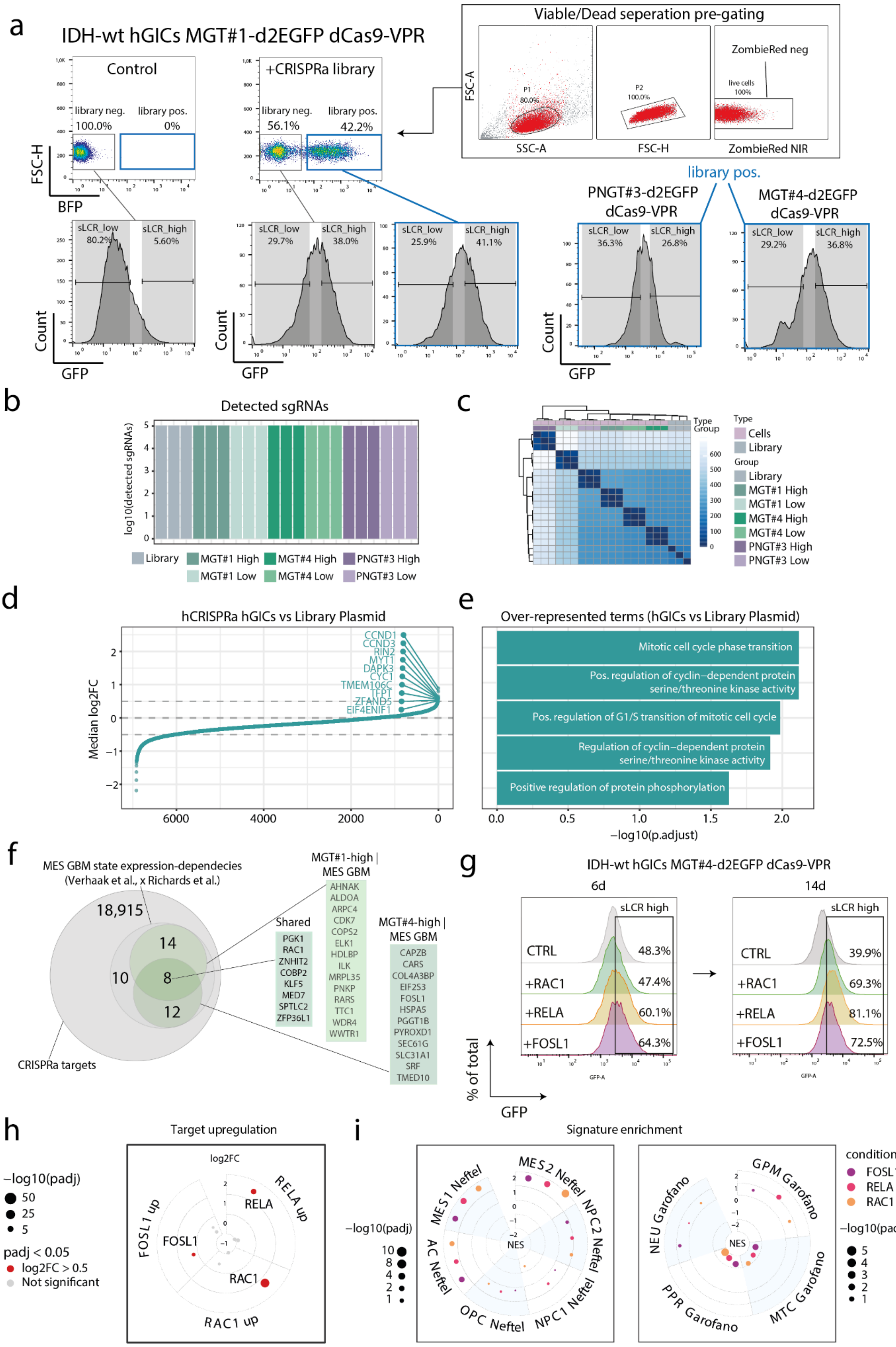

Figure S7 | Convergence of LSD, genome-wide CRISPR activation and patients' datasets towards the discovery of cell-state-specific drivers.

a) FACS gating strategy for sLCRs and hCRISPRa-v2. Note that BFP- cells (non-transduced by the genome-scale human CRISPRa sgRNA library) do also show similar levels of reporter expression, indicating that non-autonomous mesenchymal transition occurred. b) Barplot showing sgRNA reads count across the indicated conditions (color-coded). Homogeneous counts support library representation to be qualitatively maintained. C) Heatmap showing the Euclidean distance of normalized sgRNA abundance in the indicated conditions. The color legend highlights the sample origin (type) or level of reporter expression (group). D) Median log2-fold change (log2FC)-based ranking of differentially abundant sgRNAs between cells and library samples. E) Over-represented Gene Ontology terms associated with the upregulated sgRNA targets (median log2FC > 0.5) from data in (d). f) Venn diagram showing the overlap between the indicated datasets. Private and common candidate gene drivers from MGT1-high and MGT4-high screens are reported to the right. G) Representative FACS quantification of MGT4 activation by overexpression of indicated targets (color-coded) in IDH-wildtype-hGICs;MGT4-dEGFP;dCas9-VPR cells at 6- and 14-day read-outs. h) Circular dot-plot visualization of the target gene log2FC values in the differential comparisons between indicated target gene overexpressing MGT4-high cells and the unsorted control cells. Dot size and color denote significance (padj) and the significance threshold (padj < 0.05, log2FC > 0.5), respectively. i) Circular dot-plot visualization of the normalized enrichment scores (NES) for the indicated gene sets. Dot size and color denote significance of the gene set enrichment (padj) and comparison condition, respectively. FACS = Fluorescence-Activated Cell Sorting; hGICs = human glioma-initiating cells; ssGSEA = single sample gene set enrichment analysis. Source data are provided in the Source Data file.

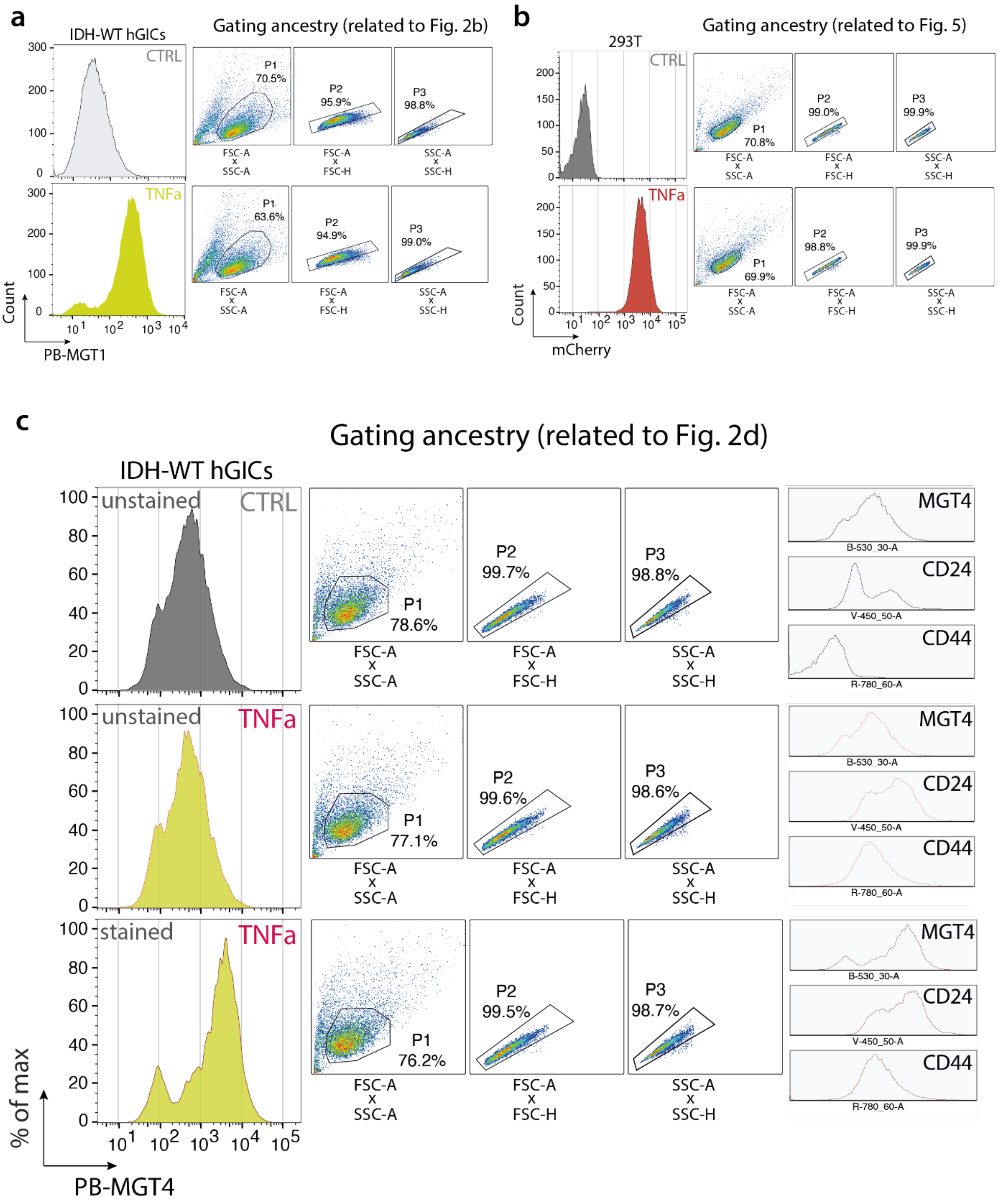

Figure S8 | FACS gating strategies.

(a) Related to Fig. 2b: Example histogram and upstream ancestry gating of untreated (grey) and TNFa-treated (lime) IDH-wildtype hGICs with PB-MGT1. (b) Related to Fig. 5: Example histogram and upstream ancestry gating of wildtype (grey) and HKGT1-transduced 293T (red). (c) Related to Fig. 2d: Example histogram and upstream ancestry gating of untreated (grey) and TNFa-treated (lime) IDH-wildtype hGICs with PB-MGT4, with or without CD24 and CD44 staining. FACS = Fluorescence-Activated Cell Sorting; hGICs = human glioma-initiating cells; TNFa = Tumor Necrosis-factor alpha; HKGT1 = Housekeeping genetic tracing sLCR #1; PB = PiggyBac.
