# Supplementary material for: Logical design of synthetic cis-regulatory DNA for genetic tracing of cell identities and state changes
Source: Nat Commun. 2024 Feb 5;15:897. doi: 10.1038/s41467-024-45069-6 (PMC10844330; doi:10.1038/s41467-024-45069-6)
Supplement: Supplementary file 3 — Description of Additional Supplementary Files [file 41467_2024_45069_MOESM3_ESM.pdf]

Description of Additional Supplementary Files:

Supplementary Dataset 1: contains inputs for sLCRs designed using LSD. The individual idenJJes are specified in the “Tab 1 - Table of Contents” and in each tab.

Supplementary Dataset 2: contains processed sequencing data associated to the CRISPR acJvaJon screens reported in Figure 6. The individual info are specified in the “Tab 1 - Table of Contents” and in each tab.
