# Supplementary material for: Logical design of synthetic cis-regulatory DNA for genetic tracing of cell identities and state changes
Source: Nat Commun. 2024 Feb 5;15:897. doi: 10.1038/s41467-024-45069-6 (PMC10844330; doi:10.1038/s41467-024-45069-6)
Supplement: Supplementary file 6 — Reporting Summary [file 41467_2024_45069_MOESM6_ESM.pdf]

nature portfolio | reporting summary

nature portfolio

Corresponding author(s): Gargiulo Gaetano

Last updated by author(s): Dec 6, 2023

Reporting Summary

Nature Portfolio wishes to improve the reproducibility of the work that we publish. This form provides structure for consistency and transparency in reporting. For further information on Nature Portfolio policies, see our Editorial Policies and the Editorial Policy Checklist.

Statistics

For all statistical analyses, confirm that the following items are present in the figure legend, table legend, main text, or Methods section.

n/a	Confirmed	
☐	☒	The exact sample size (*n*) for each experimental group/condition, given as a discrete number and unit of measurement
☐	☒	A statement on whether measurements were taken from distinct samples or whether the same sample was measured repeatedly
☐	☒	The statistical test(s) used AND whether they are one- or two-sided<br>*Only common tests should be described solely by name; describe more complex techniques in the Methods section.*
☒	☐	A description of all covariates tested
☐	☒	A description of any assumptions or corrections, such as tests of normality and adjustment for multiple comparisons
☐	☒	A full description of the statistical parameters including central tendency (e.g. means) or other basic estimates (e.g. regression coefficient) AND variation (e.g. standard deviation) or associated estimates of uncertainty (e.g. confidence intervals)
☐	☒	For null hypothesis testing, the test statistic (e.g. *F*, *t*, *r*) with confidence intervals, effect sizes, degrees of freedom and *P* value noted<br>*Give P values as exact values whenever suitable.*
☒	☐	For Bayesian analysis, information on the choice of priors and Markov chain Monte Carlo settings
☒	☐	For hierarchical and complex designs, identification of the appropriate level for tests and full reporting of outcomes
☐	☒	Estimates of effect sizes (e.g. Cohen's *d*, Pearson's *r*), indicating how they were calculated

Our web collection on statistics for biologists contains articles on many of the points above.

Software and code

Policy information about availability of computer code

Data collection	The LSD software, installation guidelines, and code are available through GitLab. https://gitlab.com/gargiulo_lab/sLCR_selection_framework
Data analysis	FACS: FlowJo (v10); Image-based screen: Harmony, Perkin Elmer; Sequence alignment: SnapGene v5.1; Pathway Analysis: Ingenuity pathway analysis (QUIAGEN)<br>Plots: R Studio ggplot2 (R v.3.6), tidyverse (v.2.0.0), readr (v.2.1.4), dplyr (v.1.1.2), RColorBrewer (v.1.1-3), pheatmap (v.1.0.12); GSEA: GSVA R package (v3.6); CRISPR screen: R (v.4.1.2), gCrisprTools (v2.0.0), MAGeCKFlute (v1.14.0), ggplot (v.3.4.2); RNA-seq analysis: R (v.4.1.2) , DESeq2 (v1.34.0), fgsea (v1.20.0), ggplot (v.3.4.2), TCGAbiolinks R package (v.2.22.4), impute R (v.1.68.0), siggenes R (v.1.68.0); scRNA-seq analysis: Seurat (v.4.3.0.1); corrplot (v.0.92); cowplot (v.1.1.1), ggpubr (v.0.6.0); sLCR transfection screen: multcomp (v.1.4-25), purrr (v.1.0.1), ggfortify (v.0.4.16)<br>LSD: python (v2.7) and R (v3.5)

For manuscripts utilizing custom algorithms or software that are central to the research but not yet described in published literature, software must be made available to editors and reviewers. We strongly encourage code deposition in a community repository (e.g. GitHub). See the Nature Portfolio guidelines for submitting code & software for further information.

Data

Policy information about availability of data

All manuscripts must include a data availability statement. This statement should provide the following information, where applicable:

- Accession codes, unique identifiers, or web links for publicly available datasets
- A description of any restrictions on data availability
- For clinical datasets or third party data, please ensure that the statement adheres to our policy

All data and codes used in this study have been deposited to the relevant repositories (https://gitlab.com/gargiulo_lab/LSD_framework). Additional protocols, data and codes, are available upon reasonable request.
TCGA data were from dbGaP database of Genotypes and Phenotypes (dbGaP) accession phs000178. The TCGA-GBM gene sets are obtained from Verhaak et al. (ref.21) and Garofano et al.(ref.23) and the GSCs dependencies were from the supplementary material in Richards et al.(ref.22). Pan-GBM single-cell data from Ruiz-Moreno et al.(ref.24) was downloaded from Zenodo (DOI: 10.5281/zenodo.6962901). CRISPRa screen data generated in this study are attached in Table S2. Tabula Muris database was accessed through https://tabula-muris.ds.czbiohub.org/ and data was downloaded from Figshare. Tabula Sapiens database was accessed through https://tabula-sapiens-portal.ds.czbiohub.org/ and data was downloaded from figshare. Gene sets for Gene Set Enrichment analysis and for pre-filtering of sLCR input were downloaded from Molecular Signatures Database (MSigDB, https://www.gsea-msigdb.org/gsea/msigdb). To define tissue-specific TF genes, we utilized data from the Protein Atlas (www.proteinatlas.org).

Human research participants

Policy information about studies involving human research participants and Sex and Gender in Research.

Reporting on sex and gender	n/a
Population characteristics	n/a
Recruitment	n/a
Ethics oversight	n/a

Note that full information on the approval of the study protocol must also be provided in the manuscript.

Field-specific reporting

Please select the one below that is the best fit for your research. If you are not sure, read the appropriate sections before making your selection.

[x] Life sciences [ ] Behavioural & social sciences [ ] Ecological, evolutionary & environmental sciences

For a reference copy of the document with all sections, see nature.com/documents/nr-reporting-summary-flat.pdf

Life sciences study design

All studies must disclose on these points even when the disclosure is negative.

Sample size

For the computational analyses and cell culture experiments in this study, a formal sample size calculation was not performed. The sample sizes were chosen based on the following considerations:

Nature of the Study:
Given the computational nature of a significant portion of our analyses and the controlled environment of cell culture experiments, the study did not involve the inherent variability associated with human or animal subjects.

Reproducibility and Consistency:
We prioritized the reproducibility and consistency of our results. The chosen sample sizes allowed for an adequate number of independent experiments or replicates to ensure the reliability of our findings.

Practical Considerations:
Practical constraints, such as the availability of resources (e.g., cell lines, reagents, computational resources), time, and laboratory capacities, were taken into account when determining sample sizes.

Statistical Robustness:
In the absence of a formal sample size calculation, we aimed for statistical robustness by including an appropriate number of replicates or data points to support the validity of our results.

Data exclusions

No data was excluded.

Replication	The sLCR screen was performed with technical replica, employing three different transfection control plasmids and conducted in three different cell lines to ensure reliability of results. HKsLCR expression was measured in three biological replica for each of the six cell lines. All attempts of replication have been successful.
Randomization	Randomization was applied in Fig. 3 in the bootstrapping analysis. Due to the nature of the remaining computational analyses, through processing of data, algorithms, or models rather than randomly assigning individuals to different conditions, randomization was not applicable. For wet lab experiments, since no animal experiments or human study participants were involved, controlled variables were used without randomization.
Blinding	Transfection/imaging and analysis of sLCR screening data have been performed by two independent scientists. Since no animal experiments or human study participants were involved, controlled variables were used without the need of scientists blind to the design. In addition, all wet lab experiments except the sLCR screen in Fig.2 were carried out by a single scientist.

Reporting for specific materials, systems and methods

We require information from authors about some types of materials, experimental systems and methods used in many studies. Here, indicate whether each material, system or method listed is relevant to your study. If you are not sure if a list item applies to your research, read the appropriate section before selecting a response.

Materials & experimental systems

n/a	Involved in the study
☐	☒ Antibodies
☐	☒ Eukaryotic cell lines
☒	☐ Palaeontology and archaeology
☒	☐ Animals and other organisms
☒	☐ Clinical data
☒	☐ Dual use research of concern

Methods

n/a	Involved in the study
☒	☐ ChIP-seq
☐	☒ Flow cytometry
☒	☐ MRI-based neuroimaging

Antibodies

Antibodies used	primary biotinylated mouse anti-human CD44-antibody (BD Biosciences #555477; 1:50) together with secondary Streptavidin APC-eFluor 780 (Invitrogen #47-4317-82; 1:100) or primary conjugated mouse anti human CD24-Vio-Blue (Miltneyi Biotec #130-126-026; 1:50)
Validation	All antibodies were used according to manufacturers' instructions and controlled with unstained cells

Eukaryotic cell lines

Policy information about cell lines and Sex and Gender in Research

Cell line source(s)	Human glioma initiating cell line (hGICs) - male - gift by Rainer Glass and Michael Synowitz (Stock et al., Nat. Med, 2012)<br>HEK293T cells - female - ATCC<br>NIH3T3 (murine) - male - Hammes Lab (MDC Berlin)<br>CHO-K1 (hamster) - Willnow Lab (MDC Berlin)
Authentication	Cell lines obtained from the ATCC or collaborators have not been authenticated by us. Our custom derived human glioma cell lines has been characterised via whole genome sequencing and regular identity checks via PCR have been performed.
Mycoplasma contamination	All cell lines have been regularly tested negative for Mycoplasma.
Commonly misidentified lines (See ICLAC register)	none

Flow Cytometry

Plots

Confirm that:

- ☒ The axis labels state the marker and fluorochrome used (e.g. CD4-FITC).
- ☒ The axis scales are clearly visible. Include numbers along axes only for bottom left plot of group (a 'group' is an analysis of identical markers).
- ☐ All plots are contour plots with outliers or pseudocolor plots.
- ☐ A numerical value for number of cells or percentage (with statistics) is provided.

Methodology

Sample preparation	Single cell suspension by Accutase dissociation of glioma spheres; filtering through cell strainer.
Instrument	BD Fortessa 5L
Software	BD FACS DIVA; FlowJo v.10
Cell population abundance	10.000 events of the final gating have been recorded and compared against each other over conditions (TNFa-treated and untreated).
Gating strategy	FSC-A vs. SSC-A to gate for live cells; FSC-A vs. FSC-H and SSC-A vs. SSC-H for doublet-exclusion.<br>Then used untreated control to set gate on the highest 5% of basal sLCR expression to determine upregulation by TNFa; the plot shows the qualitative differences.<br>Figure S8 shows the relevant gating strategy with indices referencing to the respective main figure.

[x] Tick this box to confirm that a figure exemplifying the gating strategy is provided in the Supplementary Information.
